# Direct Anastomosis of the Donor Hepatic Artery to the Supraceliac Aorta without Extension Graft during Adult Liver Transplant in the Era of Extended Criteria Donors: Report of a Case

**DOI:** 10.1155/2010/584631

**Published:** 2010-06-14

**Authors:** Jeffrey Campsen, Paul Russ, Igal Kam

**Affiliations:** ^1^Department of Surgery, Division of Transplantation, University of Colorado Denver, Campus Box C-318, 1635 Aurora Court, Aurora, CO 80045, USA; ^2^Department of Radiology, University of Colorado Denver, Aurora, CO, USA

## Abstract

Arguably, one of the most challenging aspects of liver transplant surgery is the hepatic artery reconstruction. When the donor and recipient arteries are normal, this anastomosis can still be difficult. However, when the recipient artery has been dissected or is small other alternative reconstructions must be considered. Routinely, the donor surgery includes removing the iliac artery and vein specifically to aid in alternative reconstruction techniques. With the increase use of extended criteria donors (i.e., specifically age >55) the iliac vessel may be unusable because of atherosclerotic disease. This paper describes revisiting an alternative technique for hepatic artery reconstruction during cadaveric liver transplant when the recipient artery has been dissected and the iliac vessels were unusable secondary to arterial plaque from a 75 yo donor. Herein, we describe the successful anastomosis of the celiac artery with aortic patch from the donor directly to the supraceliac aorta of the adult recipient.

## 1. Case Report

A 68-year-old female with primary biliary cirrhosis received a liver transplantation from a deceased heart-beating donor for end-stage liver disease with a MELD of 24. Preoperative study showed no evidence of hepatic artery abnormalities. The donor was a 75-year-old female that was pronounced brain-dead after a stroke. The donor's only comorbidity was significant history of cigarette use. During the donor surgery the celiac artery did not have any atherosclerotic disease and the hepatic arterial anatomy was normal. Of note, the iliac arteries were severely diseased with hard circumferential plaque. 

**Figure 1:**
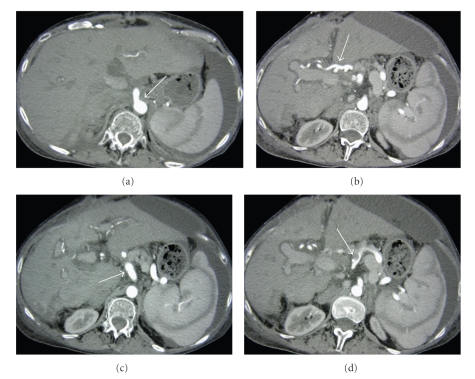
(a) Axial source image from CT angiogram (CTA) demonstrates donor hepatic artery anastomosed to the proximal aorta (arrow). Note normal arterial perfusional striations in the spleen. (b) Axial CTA source image shows the donor hepatic artery coursing to the hepatic hilum (arrow). (c) Axial CTA source image shows thin, transversely oriented intimal flap from an arterial dissection in the recipient celiac trunk (arrow). (d) Axial CTA source image depicts the initmal flap of the celiac trunk dissection extending into the splenic artery. False lumen thrombus in the partially occluded splenic artery is apparent (arrow).

Liver transplantation was performed by using the Piggy-back technique with restoration of vena caval and portal vein blood flow before the arterial reconstruction. It was then discovered that the native hepatic artery was dissected (Figures 1(c) and 1(d)). In this situation we routinely use a cadaveric iliac jump graft to the infrarenal aorta to reconstructe the hepatic artery; however, the donor's vessels were unusable [1]. The decision was made to anastomose the donor celiac artery with aortic carrel patch directly to the supraceliac aorta (Figures 1(a) and 1(b)).

The length of the donor celiac artery was adequate without tension. The gastrohepatic ligament had already been ligated during the hepatectomy and the stomach was retracted laterally. The diaphragmatic cura were divided in a muscle splitting fashion in an attempt to preserve them starting just below the insertion of the aorta through the diaphragm. The supraceliac aorta was exposed inferiorly for approximately 3-4 cm which allowed cross-clamping with two straight vascular clamps above the celiac artery origin. We did not dissect out the aorta circumferentially to avoid the posterior lumbar arteries in that area of the aorta. An arteriotomy was made and enlarged with two 4.8 mm aortic punches. The anastomosis was created with 5–0 prolene in running fashion. Cross-clamp was 17 minutes. 

**Figure 2:**
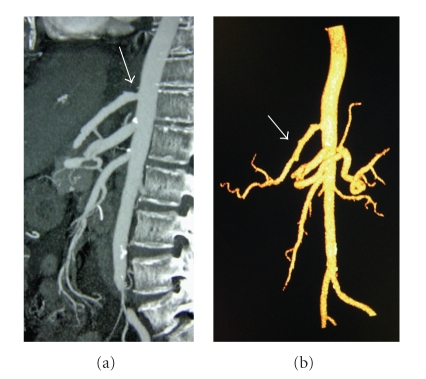
(a) Thin section two-dimensional sagittal CTA reconstruction of the aorta shows the anastomosis (arrow) of the hepatic artery to the proximal abdominal aorta superior to the celiac trunk and superior mesenteric artery (SMA). (b) Colorized three-dimensional volume rendered obliquely oriented CTA reconstruction shows the course of the donor hepatic artery (arrow) from the aorta to the proper hepatic artery bifurcation.

Postoperatively, the patient's creatinine rose to 1.6 mg/dL on POD1 and returned to a baseline level of 0.8 mg/dL by POD4. Transaminases reached 1000 (U/L) on POD1 and came down immediately. They did not seem affected by the arterial reconstruction and were probably a result of the age of the donor. The patient was started on TPN from POD1 to POD 9 and had a nasogastric tube until POD 2. TPN was used as a precaution because we were concerned that the patients' age combined with ischemia time to the gut from a supraceliac aortic clamp would cause a significant ileus. She did have some delay in bowel function. We left her nasogastric tube in for two days and then advanced her diet slowly over the next few days. During this time we left her on TPN until she had significant caloric intake by mouth. She tolerated a regular diet on POD 9. She had no evidence of neurological dysfunction [2]. The arterial anastomosis was patent by CT angiogram on POD 9 (Figures 2(a) and 2(b)).

Both the short and long-term outcomes are excellent. She is now a year out and has had no complications including no episodes of rejection. The graft is functioning well and she did not need a retransplant.

## 2. Discussion

Successful hepatic artery reconstruction can normally be achieved with the donor celiac artery anastomosied to the recipient hepatic artery. Occasionally, the recipient hepatic artery is insufficient to provide arterial inflow to the graft. At our center, in these cases, we will use the iliac artery from the donor as a jump graft to the infrarenal aorta with good success [3]. However, in this case the iliac arteries were damaged by severe atherosclerosis. With increasing use of extended donor criteria, in this case, an age of 75 years, poor quality of the iliac vessels may be encountered. In this situation, directly anastomosing the donor carrel patch to the supraceliac aorta is a viable alternative. Of course, this is a last resort before attempting use of an artificial conduit which has its own risk of nonhealing and infection in the face of immuosuppression.

Unfortunately, dissection of the hepatic artery can occur and is a difficult problem to overcome. In this patient, there were no preoperative risk factors such as TACE (transarterial chemoembolization), hypertension, or vascular disease, except age. The reason for the dissection then could have been technical or from positioning of a retractor during the anhepatic phase of the transplant. Once the dissection was recognized, the team immediately began determining options for arterializations, realizing that the donor iliac vessels were unusable and there were no compatible donor iliac vessels from previous transplants in storage. Because the donor celiac artery was lying on the aorta in perfect position we decided to perform the reconstruction described above. The main issue was dissection of the cura of the diaphragm. The previous hepatectomy had moved the stomach and exposed the cura. We preformed a muscle splitting dissection to preserve the cura. Then we placed two straight vascular clamps on the aorta. After the anastomisis was complete, the cura surrounded the reconstruction. 

Two case series in the early 1990's described using the supraceliac aorta as arterial inflow to a liver graft. In Shaked et al. they preformed a primary end to side common hepatic artery and supraceliac anastomosis without the use of grafts in eleven adult patients mainly for small arteries <3 mm or for hepatic artery thrombosis [4]. Hennein and colleagues described this anastomosis in one adult patient for retransplantation. Both of these reports have small numbers in adults, but, demonstrate positive outcomes [1]. This approach is not completely without risk, and cross clamping of the aorta can significantly affect both renal and bowel function because of the ischemic time placed on these organs. However, based on the above data and our experience there does not appear to be an increased risk for hepatic artery thrombosis in this reconstruction. 

While the modest experience thus far is generally good, direct anastomosis to the supraceliac aorta with the donor's aortic carrel patch should not be forgotten as an alternative anastomosis when the recipient and donor's anatomy dictate. With the increased use of older donors the iliac vessels are often not usable. There is a definite increased morbidity with this reconstruction, but, it can be performed successfully to provide adequate arterial inflow to liver grafts in difficult situations.
